# A Computerized Continuous-Recognition Task for Measurement of Episodic Memory

**DOI:** 10.3233/JAD-190167

**Published:** 2019-05-21

**Authors:** J. Wesson Ashford, Franck Tarpin-Bernard, Curtis B. Ashford, Miriam T. Ashford

**Affiliations:** a Department of Psychiatry & Behavioral Sciences, Stanford University School of Medicine, Stanford, CA, USA; b War Related Illness & Injury Study Center, VA Palo Alto Health Care System, Palo Alto, CA, USA; c HAPPYneuron, SAS, Lyon, France; d MemTrax, LLC, Redwood City, CA, USA

**Keywords:** Aging, Alzheimer’s disease, cognition, cognitive impairment, dementia, episodic memory, memory, reaction time, recognition

## Abstract

Based on clinical observations of severe episodic memory (EM) impairment in dementia of Alzheimer’s disease (AD), a brief, computerized EM test was developed for AD patient evaluation. A continuous recognition task (CRT) was chosen because of its extensive use in EM research. Initial experience with this computerized CRT (CCRT) showed patients were very engaged in the test, but AD patients had marked failure in recognizing repeated images. Subsequently, the test was administered to audiences, and then a two-minute online version was implemented (http://www.memtrax.com). The online CCRT shows 50 images, 25 unique and 25 repeats, which subjects respectively either try to remember or indicate recognition as quickly as possible. The pictures contain 5 sets of 5 images of scenes or objects (e.g., mountains, clothing, vehicles, etc.). A French company (HAPPYneuron, SAS) provided the test for 2 years, with these results. Of 18,477 individuals, who indicated sex and age 21–99 years and took the test for the first time, 18,007 individuals performed better than chance. In this group, age explained 1.5% of the variance in incorrect responses and 3.5% of recognition time variance, indicating considerable population variability. However, when averaging for specific year of age, age explained 58% of percent incorrect variance and 78% of recognition time variance, showing substantial population variability but a major age effect. There were no apparent sex effects. Further studies are indicated to determine the value of this CCRT as an AD screening test and validity as a measure of EM impairment in other clinical conditions.

## INTRODUCTION

One of the great challenges of modern Psychology and Medicine has been the assessment of memory function. Memory function is required in nearly all human endeavors and its dysfunction is a hallmark of aging and many common and uncommon diseases, in particular Alzheimer’s disease (AD) [1]. Memory is a complex process which involves extensive sensory, association, and executive networks of the brainstem and cortex for encoding, recognizing, and reconstituting information [2]. In memory function, information is perceived, then stored (learned) for possible later usage (remembering). Stored information is accessed through recognition and active retrieval processes (e.g., free recall). The perception of information requires the activation of complex circuits which have developed throughout life, and memory encoding mechanisms modify these same circuits to store new information [3].

Episodic memory (EM), the function of storing information in a form which can be retrieved after distraction, is frequently disrupted by brain dysfunction and is particularly difficult to quantify. Assessment of EM has generally involved using rudimentary tests such as the recall of a list of words (e.g., Buschke Selective Reminding Test [4]) or reproduction of a modestly complex figure or figures (e.g., Benton Visual Retention Test [5]). However, such tests of EM function have a limited dynamic range. Though memory tests are enormously complex and evaluate numerous processes involving learning, storing, and retrieval of information, they cannot assess any of these individual functions precisely or reliably. Further, various disorders of memory, ranging from mild through profound, are also variable in how much impairment they cause in each memory processing component. Common clinical tests of memory usually provide only a qualitative indicator of memory dysfunction, complicated by the individual execution and interpretation of the psychometrician. For example, in the fields of dementia and AD, there is a large list of tests which have been used for cognitive assessments and screening [6]. In spite of numerous studies of relevant cognitive tests [7–9] and the development of test composites [10–15], the inadequacies of screening and severity assessments are likely factors which have hampered advancement in the AD field. Many investigators have also developed computerized cognitive tests for dementia and AD [6, 16–19], though most such tests are simple implementations of commonly used neuropsychological tests. One test uses a computerized version of paired associate learning [20]. New directions using computerized continuous recognition tasks (CCRTs) and fMRI (functional MRI) are being used to further the understanding of the medial temporal lobe and memory [21, 22], which are both of central issues in AD. Therefore, there is an important need for approaches which can assess EM function rapidly with greater precision and standardization [23].

In consideration of improving the assessment of EM and the function of those systems which are most relevant to human memory disorders, it is necessary to reference those processes associated with activation of temporo-parietal regions of the neocortex and the hippocampus [24, 25]. These structures are targeted by many brain imaging approaches which examine human memory, and such studies, including fMRI, show that memory encoding is an activator of these areas. The most specific activation of these brain regions is done using continuous recognition task (CRT) paradigms [26–30]. Such memory paradigms are most readily implemented using computer programs.

With the advent of computer technology, refinement of memory testing methodology and analysis can now use computer and internet technology to provide more precise measures of memory function across normal populations, aging, and many central nervous system disorders. Such developments have the potential to far surpass paradigms used by traditional neuropsychological testing. Findings using CRTs suggest that there could be a potentially important role for online CCRTs for testing subjects for clinical and research purposes, as well as for screening and monitoring memory encoding. Further, such testing could be used in the dementia and AD fields as a screening and assessment measure to initiate dementia assessment and diagnostic tests and contribute to the determination of the severity of the cognitive impairment [31], leading to estimation of severity of the underlying pathology as well as the social dysfunction [10], and thereby guide clinician care [32]. And a quick, precise, widely available, and inexpensive test could have widespread applications for any area in which memory function is a concern, but particularly in the field of AD.

A CCRT provides a precise assessment of EM function, which is the specific type of memory most selectively affected by AD, and this type of memory begins showing deterioration early in the course of the development of Alzheimer-type dementia [33, 34]. A CCRT test of EM has potentially widespread applications because this type of memory is vulnerable to a variety of other conditions, including traumatic brain injury, hypoxia, hypoglycemia, certain vitamin deficiencies, and numerous neurotoxins, including ethanol and cannabinoids, as well as the effects of anesthesia and cancer chemotherapy [35]. The question raised here is whether a CCRT can provide adequate precision and accuracy for EM assessment with respect to age to be a potentially convenient and useful clinical tool.

In a prior study, a CCRT slide presentation was administered in an audience setting, allowing audience members 5 seconds to indicate on a sheet of paper whether they had seen a picture before in the series [36]. In the results of testing of over 1,000 individuals, an age effect on correct recognitions in visuo-spatial memory performance was clearly shown. In the current study, the previously-described audience CCRT was adapted to be administered online over the internet, using a 3-second viewing of images, additionally measuring response time, with immediate presentation of the next image. In this implementation, performance was measured as both incorrectness of recognition and recognition speed (reaction-time). The purpose of the present analysis was to determine the average performances of incorrectness and recognition speed with respect to age and gender and the statistical variation from the average performance (standard deviations) with respect to age for this population sample. Such information will support the use of online testing for inexpensive cognitive screening, identification of potential cognitive impairment, and tracking cognitive function both for individuals at risk for developing impairment and monitoring change over time.

## MATERIALS AND METHODS

### Test paradigm

The CCRT employed in this study was first developed in a dementia clinical setting, where it was well tolerated by patients and also tried by interested caregivers. Test images were poorly recognized by individuals thought to have dementia of the Alzheimer-type, but those considered to have frontotemporal dementia responded to many stimuli regardless of whether they were repeated images. This test was adapted for administration to audiences using paper & pencil and a PowerPoint slide show timed to show 50 images, one every 5 seconds [36]. That test format, providing a sequence of 25 unique and 25 repeated (5 of the repeats being second repeats and groups of 5 unique images being chosen from each of 5 conceptual categories), was also implemented to an online website (URL: http://www.memtrax.com). Between September 2011 and August 2013, this test was hosted by HAPPYneuron, SAS, a French company. Individuals participating in the HAPPYneuron program (http://www.happyneuron.com) were specifically solicited to participate in the testing program, with a new version of the test being recommended by email and available to participating subjects every month. After a subject downloaded the program, an opening screen introduced the user to memory function and reasons to monitor one’s own memory and offered an opportunity to register at the website, including providing birthdate, gender, and an email to receive a reminder to take the test with a new series of pictures each month. When the test was downloaded, the user’s computer showed a single screen instruction to look at each of 50 pictures carefully and try to remember them and to press the space-bar of the computer as quickly as possible whenever a repeated picture appeared. The user was instructed to press the space-bar to begin viewing the series of 50 pictures. Each picture was shown on the screen for 3 seconds or until the space-bar was pressed, at which time the next picture was shown immediately.

The picture sets, used for all subject performances reported here, included photographs of objects, including structures, kitchen items, furniture, landscapes, and bodies of water. The images were shown in a specific order in which the first 3 pictures were new, but the following pictures were repeats shown in a pseudo-random order, with no more than 4 repeats or 4 new pictures being shown in a row. Response time, measured using the internal clock of the local computer, was recorded for every image, with a full-time measure (e.g., 3 seconds) indicating no response. Response times less than 300 ms were also interpreted as “no response”, jumping immediately to the next picture after 500 ms. However, after any response (correct or incorrect), the next picture was shown immediately (after at least 500 ms). Data collected was the response time to each of the 50 items presented. Analytic calculations were performed immediately indicating percent correct (true positives and true negatives) and recognition time for responses to repeated images. Subjects were provided with these measures without interpretation or consequential recommendations.

In the present study, a short version (15 items) of the test was provided for practice before subjects registered to take the full test for the first time. The reason why this practice test was provided is that in audience testing, approximately 10% of the subjects did not understand the instructions, but a practice test appeared to remedy this.

Raw data was reduced by considering the response to each stimulus. A response was recorded if the reaction time was between 300 and 2900 ms. Responses were counted as correct (true-positives or hits) for repeated items and incorrect (false-positives or false-alarms) for first presentations. Accordingly, reaction times less than 300 or longer than 2900 ms were considered non-responses (incorrect for repeated items and correct for first time presentations; note that responses less than 300 ms would not represent a physiological response to a perceived stimulus and correct recognition responses nearly always occurred in less than 2000 ms and so 2900 to 3000 ms responses were considered non-recognitions). Percent of errors was calculated by adding the misses and false-positives, multiplied by 2 (50 image presentations). The reaction times for the correct responses (to repeated items only) was averaged and considered to be the mean recognition time for that individual. Performance of less than 60% correct, making 10 or more false-positive responses, or responding correctly to less than 15 of the repeated images was considered a failed test. The basic two performance measures analyzed for each participant successfully performing the test were the percent of incorrect responses and the mean recognition time for the correct responses.

### Subject selection

Data for each user completing the test was stored on a server located in Lyon, France (HAPPYneuron, SAS). Subjects were initially asked to sign up for an account for a small fee. After 4 months, the registration was provided for free for individuals providing a minimum amount of information: birth date, gender, employment status, preferred language, and four general health questions. There was no method to verify any information, though users were permitted to provide a link to prior registration with HAPPYneuron, thus not remaining anonymous. Permission was granted by the Human Subject Protection Committee of Stanford University to provide such a test to subjects anonymously [36], then later to review anonymous data provided from France. The Stanford IRB approved analysis of the test results only for individuals over the age of 21 years.

### Subject selection for detailed analyses

Complete data were recorded for the 30,435 times the test was taken between September 2011 and August 2013. For the present analysis 4,800 tests were eliminated for being taken after the first time a registered user took the test (repeated tests). The test was taken between 2 and 23 additional times among the over 25,000 participants, indicating that many individuals who took the test willingly once took it repeatedly (repeat tests were not further analyzed here).

The subjects selected for analysis in this study were all those who registered and took the test, presumably taking the test for the first time, and the data examined were from that first taking of the test after registration, 25,635 individuals. Of these registrants, 4,999 individuals provided birthdates suggesting that their age was under 21 at the time of the first test, so those individuals’ data were excluded from analyses.

Since the subjects were unidentifiable individuals entering the site to take the test from anywhere in the world, a difficult issue was to determine the likelihood that the provided data by a subject was reasonable. Consequently, data were eliminated from subjects recording unlikely birthdates (before 1/1/1910; earliest remaining 12/1/1912), over 100 years of age (oldest 99.88 years), or not providing gender, a total of 1,574 individuals, bringing the total number of available tests to 18,974. Of these subjects, 407 individuals (115 males and 292 females) made no responses. Also, 26 males and 64 females made between 1 and 5 total responses indicating a very low level of responsiveness and a recognition rate of repeated images of 10% or less. Also, 24 men and 41 women made between 6 and 10 total responses. The 497 individuals with 5 or fewer responses were eliminated from further analysis as not appropriately taking the test, leaving 18,477 subjects for further analysis.

In examining the results for those 18,477 subjects, 1,203 tests showed performance of less than 70% correct recognitions. A more difficult criterion is maximum recognition time of 1800 ms, which was chosen because only 34 (0.2%) of the remaining individuals responded so slowly (only 9 of them were over 70 years of age). The average age of the individuals slower than 1800 ms was 58 years, while the average for the remaining group was 50 years of age, so this limit was not considered to be a major age-related factor or to significantly affect the data analysis. In this group, 470 subjects had performance levels of less than 60% correct and/or average reaction times over 1800 ms (2.5% of this population). Such measures were considered inadequate test performance for unknown reasons (poor comprehension of the test, visual problems, lack of interest, not paying attention, etc.), and there is no indication of the causes of this level of performance. Eliminating these 470 individuals, 2.5% of 18,477, left 18,007 subjects, who took the test for the first time, indicated gender and an age between 21 and 99 years, and made at least 6 responses on the test, for detailed analysis. For these individuals, 5,665 individuals (32%) indicated that they were male, and 12,342 (68%) indicated that they were female. The mean age of the group was 50 years (SD = 15, Range 21–99 years), for males, 51 years (SD = 17), for females, 49 (SD = 14).

The subjects were asked to choose their language. In this group of 18,007 individuals, 65% chose their language as French (divided as 17% males and 48% females), and 35% chose their language as English (divided as 15% of the males and 20% females), suggesting that most of the individuals taking the test lived in France. An additional question was asked of the subjects as to whether they had a memory problem. In the group, 92.9% % of the individuals answered this question as negative (“no” or “non”) or positive (“yes” or “oui”), 89% of the males (44.5% answering negative and 55.5% answering positive) and 94% of the females (38.5% answering negative and 61.5% answering positive). Subjects were also asked their employment status (see Table 1). Other questions, including medication use, alcohol use, sleep issues, and medical conditions were also asked. These answers and their implications were not further considered in this analysis. There was no question about years of education.

**Table 1:** Population answering gender and date of birth questions in range, according to selected language and employment status (numbers in percent by each row)

Language	Gender	Percent	Percent Answering	Employed Full Time	Employed Part-Time	Retired	Unemployed	Average Age (y)
French	Male	26.6	93.6	45.4	6.3	30.0	11.8	50.9
French	Female	73.4	95.4	36.7	14.1	27.8	16.9	47.4
English	Male	41.9	83.0	46.9	7.1	18.4	10.6	51.2
English	Female	58.1	89.3	40.1	16.7	19.1	13.4	49.4
Both	Male	31.9	88.8	51.9	7.5	27.8	12.7	51.1
Both	Female	68.1	93.6	40.3	15.9	27.0	16.9	48.6
French	Both	65.3	94.9	41.1	12.7	29.9	16.3	49.3
English	Both	34.7	86.3	49.5	26.2	10.2	14.1	50.7

### Data analysis for individual meeting defined performance criteria

Given the large number of both male and female subjects, data were analyzed separately for individuals indicating gender as male or female, and the number of subjects was analyzed for each year of age. The total number of responses and number of correct responses was determined, as well as the cumulative number of correct responses by percent correct. Percent incorrect and recognition time were analyzed as a function of age. In examination of the distributions of all responses, the exclusion parameters eliminated all subjects with average response times shorter than 558 ms. To determine whether there was a speed/accuracy trade-off, recognition time was plotted versus percent correct. To estimate where age-appropriate performance ranges might be, analyses were made by age for number wrong, +1STD, and +2STDs and recognition time, +1STD, and +2STDs versus age.

All statistical analyses were carried out using EXCEL (Microsoft Corporation).

## RESULTS

### Population gender and age

**Fig.1:**
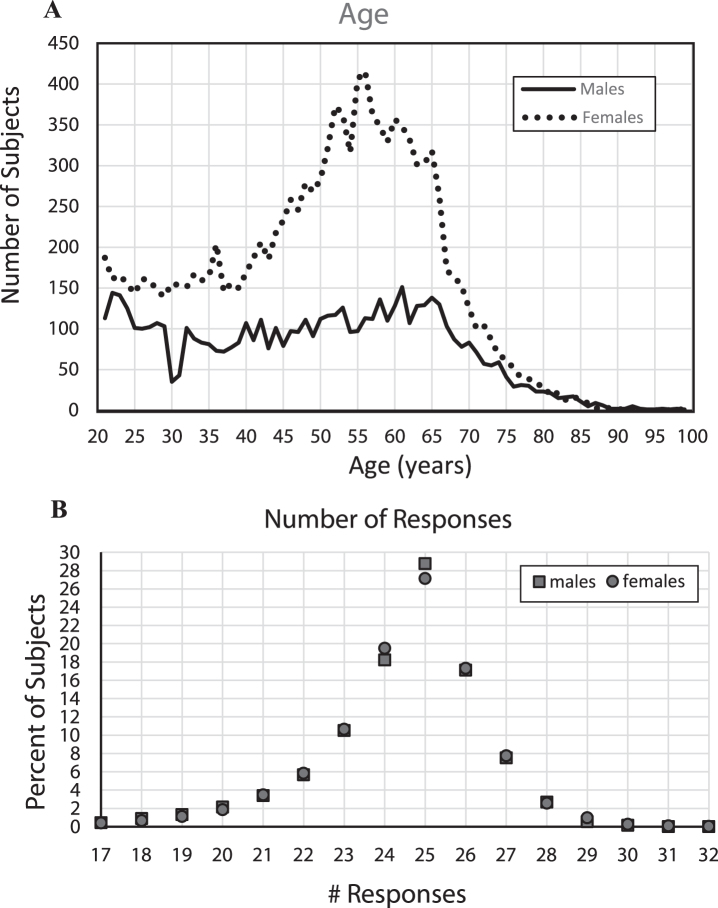
A) Number of subjects by year of age, B) Percent of subjects providing each number of responses.

For these analyses, the included group of 18,007 individuals had a reasonable range of performances on each metric: number of total responses, correct hits, correct rejections, total incorrect responses, and mean recognition reaction time. All results for males and females were analyzed separately to determine whether any significant gender differences were present and to show the stability of the measures. There were more than twice as many female subjects (12,343, 68.5%) as male subjects (5,665, 31.5%), a result not related to population age distribution since there tend to be more males in similar populations before age 45 years; there was essentially no difference in the numbers in this sample after age 80 (Fig. 1), when the number of females becomes more than twice the number of males in most Western populations. Of note, a substantial proportion of females indicated ages between 40 and 70 years of age, which accounted for the largest group difference between the number of males (3,269, 57.7% of all males) and females (8476, 68.7% of all females). Though over twice as many females took the test as males, on all metrics and comparisons, male and female performances had no significant differences and were remarkably similar.

With the large number of subjects, stable performance metrics could be estimated for each year of age between 21 to 85 years. However, since the proportion of individuals taking the test at each age was not uniformly distributed (Fig. 1), it was necessary to adjust the data analyses for age. Despite the large number of subjects in the full sample, due to the progressive decrease in the numbers of subjects per year of age after age 65, performance metrics could not be made reliably for individual year-of-age groups over 85 years. The average number of individuals for each year of age from 21–75 for males was 98 and for females was 219. The number of subjects at each year of age over 75 years decreased substantially with age relative to the number of younger individuals taking the test, with 216 males between 76–85 (averaging 11 per year of age) and 273 females in this range (averaging 14 per year of age). Then, there were only 41 males between 86 and 99 and 25 females in this age range. With the substantial increase of variability of performance with age, the precise statistics of the older age ranges are poorly estimated with this small population.

### Analysis of incorrect responses

**Fig.2:**
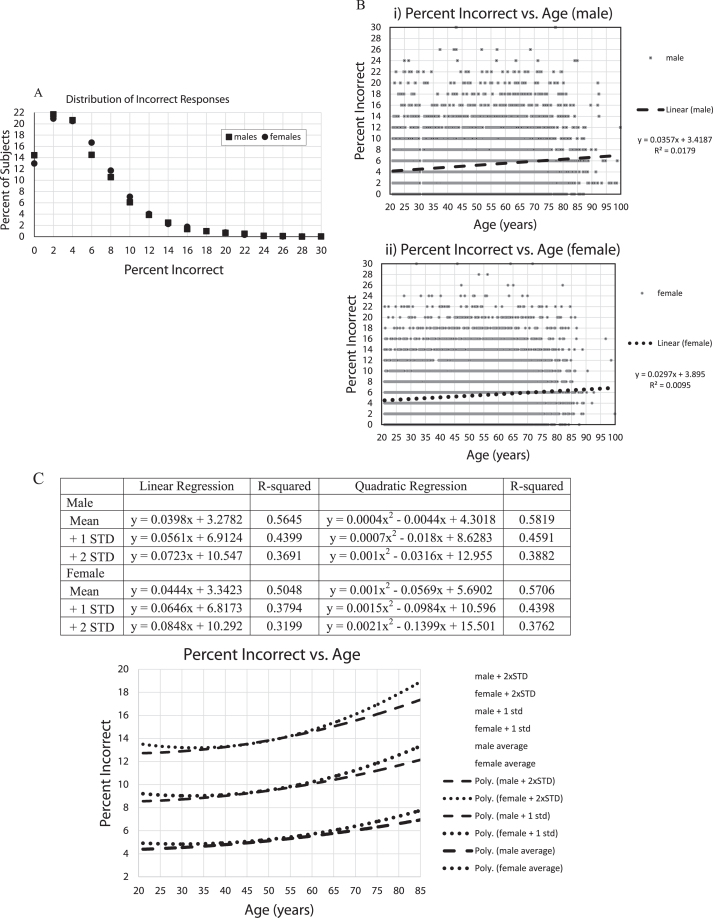
A) Percent of subjects versus percent incorrect. The plots are essentially the same for males and females. About 35% of subjects missed none or 1 image. About 45% erred on 3 or more. 12% errors = 1.5 standard deviations; 16% errors = 2.0 standard deviations; 20% errors = 2.5 standard deviations; 24% errors = 3.0 standard deviations, B) i,ii Percent of incorrect responses for all subjects, 5,665 males (i), 12,342 females (ii), with linear regression lines and linear equations,C) Percent of incorrect responses were averaged for each year of age by gender with +1 and +2 standard deviations calculations for each age, trendlines for each Male and Female calculated by EXCEL spreadsheet graph. Table shows Male and Female, Mean, +1 standard deviation +2 standard deviations, linear regression curves, R-squared, quadratic regression lines, R-squared. Graph shows: Male: dashed lines; Female: dotted lines; quadratic trendlines, lower are for the average of each year of age, with +1 STD lines above them, and +2 STD lines above that.

In examining the total number of responses for each subject (Fig. 2A), the largest percentage of responses was exactly 25 (50%) which is incidentally the number required for 100% correct performance. There were in general fewer than 25 total responses across the participants, a non-random distribution, suggesting that there was a greater tendency for subjects to not recognize images as repeats than to over-respond by indicating that an image that was not seen before was a repeat. The tendency was to make between 18 and 24 responses, indicating that after removal of subjects who had exclusionary performances (chance, very few, too many, or recognition times out-side the credible range), most of the remaining total responses were in a relatively narrow range, supporting the legitimacy of the performance metrics of the sample population reported here.

For the number of incorrect responses (Fig. 2B), of the individuals with acceptable performance, the average percent incorrect was 5.2% (2.6 errors) (std = 4.5%) and 5.4% (2.8 errors) (std = 4.4%), respectively for males and females. Though performance measures of more than 30% incorrect were excluded, only 11% of each group scored more than 10% incorrect (5 errors), and there were very few individuals who made between 20% (10 errors) and 30% (15 errors) incorrect responses, only 1.7% of the males and 1.4% of the females. Thus, general performance was good, though fewer than 15% of the subjects had perfect scores and fewer than 22% made exactly 1 error, indicating a modest ceiling effect for the test (see Fig. 2B for specific standard deviations).

In examining the percent incorrect versus age (Fig. 2C, D), there was an increase in the number of incorrect responses with increasing age. However, the age effects accounted for less than 2% of the variance (note: R-squared is considered to represent the proportion of the variance explained by the regression equation) in total incorrect responses, even using quadratic regression (Fig. 2D). In looking at the raw data, there was little obvious difference between individuals under 80 and over 80 years of age. In examining the means and standard-deviations of the responses for each year of age, thus, balancing out the variation in the number of individuals taking the test at each age, there remained an increase of errors with age, explaining over 50% of the variance (Fig. 2D). Further, there appeared to be a steeper increase of incorrect responses per year of age over the age of 60 years, accompanied by an increase of population variance with age.

### Analysis of recognition time

**Fig.3:**
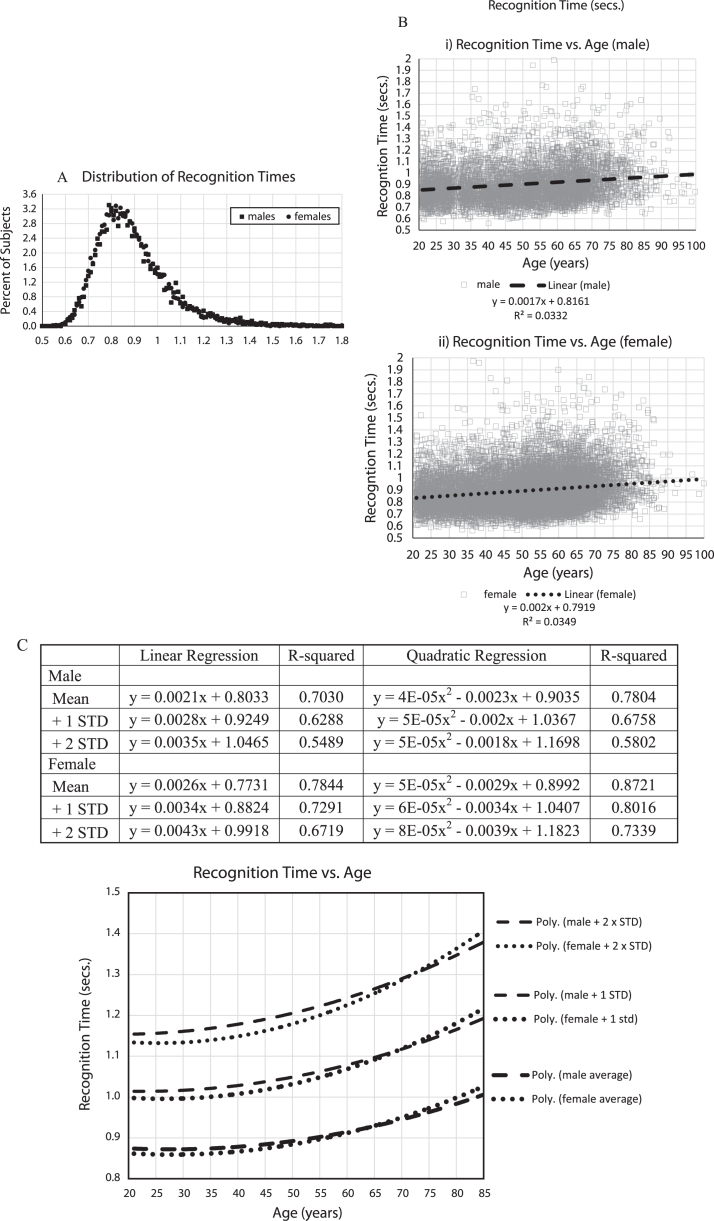
A) Percent of subjects versus mean recognition times, B) i,ii Mean response times for all subjects (seconds), 5,665 males (i), 12,342 females (ii), with regression lines and linear equations, C) Mean response times (seconds) averaged for each age by gender with +1 and +2 standard deviations calculations for each age. Table shows data from EXCEL spread sheet calculations for trendlines for Male and Female, Mean, +1 standard deviation, +2 standard deviations, linear regression curves, R-squared, quadratic regression lines, R-squared. Graph shows: Male: dashed lines; Female: dotted lines; quadratic trendlines, lower are for the average of each year of age, with +1 STD lines above them, and +2 STD lines above that.

Regarding the recognition time distribution (Fig. 3A), of the 18,007 included participants, based on specific exclusion criteria, there were no response times faster than 558 ms. The average recognition time was 902 ms (std = 161 ms; median = 872 ms) for males and 893 ms (std = 153 ms; median 868 ms) for females, with no suggestion of a difference in the overall distribution. The distribution of recognition times was skewed with a decreasing number of individuals responding in over 1.5 s.

Analyzing recognition time versus age (Fig. 3B, C), mean response times increased with age more than the percent incorrect, with age effects accounting for between 3 and 4% of the variance (Fig. 3B). In examining the means and standard-deviations of the responses for each year of age, balancing out the variation in the number of individuals taking the test at each age, similar to the percent of incorrect responses, there was a substantial increase of recognition time with age, explaining 70% of the variance for men and 78% for women, and there appeared to be a similar steeper increase over the age of 60 years (Fig. 3C). There was also an increase of population variance with aging.

### Analysis of speed versus accuracy

**Fig.4:**
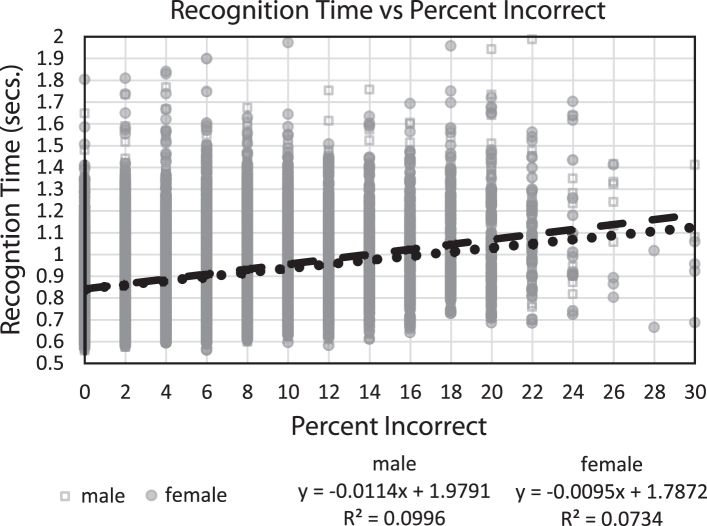
Plot of mean response times (seconds) for every subject versus percent incorrect, with linear regression lines and equations, respectively.

In comparing recognition time with percent correct (Fig. 4), the relationship between speed and accuracy accounted for 10% of the variance for both genders, with individuals who performed more correctly having faster recognition times. Thus, across individuals, there was no speed/accuracy trade-off; in fact, the opposite was the case. Since only one test session was analyzed for each subject, the occurrence of a speed/accuracy trade-off phenomenon for individuals could not be analyzed to determine if this factor was present in individual performances.

## DISCUSSION

### Online testing of a population sample

The primary purpose of this study was to determine whether a substantial number of individuals would try an online test of memory function voluntarily, and the results presented here show that many subjects did participate in this computerized assessment and provided reasonable demographic information consistent with expectations of the sampled population. The secondary purpose was to evaluate whether participants performed the computerized test did so in expectable ranges, and more than 90% of participants taking the test had response ranges indicating reasonable effort. The third objective was to estimate the statistical parameters of performance on the test, including incorrectness and recognition speed, and the responses of the participants engaged in the test were likely a valid reflection of each participant’s capability. Additional issues were to determine the relationship of performance metrics with respect to age and gender and the statistical variation from the average performance (standard deviations) with respect to age for this population sample.

Of interest, the number and distribution of individuals taking this test indicated that there are many people who are concerned about their memory. The largest group taking with test was females from ages 40–70 years, reflecting the common concern about memory among women related to menopause. This group may also have a fear of developing AD, which is currently the second leading cause of death for women over the age of 75 years in the US (CDC – Centers for Disease Control, 2016 data).

The reasons for the decreased number of individuals in the older age range could be due to the decreasing number of people in this age range, decreased interest in self-assessment or memory testing, or unfamiliarity with or limited access to computers, issues relevant to interpretation of the data but not resolvable with the population participating in this study.

### Validation of performance: responses and recognition time

The first issue in the analysis of this data set was to determine what responses of a subject indicated that this individual understood the test instructions and tried to perform the test online as well as possible. It was important to determine individuals who either did not understand the test directions, did not press the space bar (the response indication), or did so 15 or fewer times, for unknown reasons, so that they could be eliminated to reduce the impact of these individuals on the analyses. Such responses are no more indicative of cognitive performance than chance occurrences, though any poor performance may indicate other issues (e.g., distraction or sensory impairments such as visual difficulty). Of the 18,477 individuals in the age range 21–99 years and making at least one response on the test, 97.5% performed within a reasonable, non-chance range of correctness and speed of response, suggesting that anonymous individuals taking the test online perform the test with appropriate attention to the task and at levels potentially acceptable for inferring precise levels of cognitive function.

For the 18,007 individuals selected for reasonable performance, the range of errors and reaction times were analyzed and found to have appropriate statistical distributions. Remarkably, across the years of age from 21 to 75, for each year of age, there was a comparable distribution of performances for both men and women, showing that the data were highly stable. The changes with age followed the expectable patterns of slight decline, while in the older age ranges, over 75, the rate of decline increased but so did the variability, as is expected with aging. The increase of variance of percent incorrect with age was similar to the data seen with the audience version of the task [36]. The increase of variance in recognition time with aging is also what is generally expected with increasing age.

It is well known that there is a general decline of cognition, particularly memory, with age [37, 38]. Age-related impairment in a CRT examining recognition memory has also been described previously [39]. The results of the analysis of this group of subjects, both percent incorrect and recognition time, is consistent with this general finding in the literature [40]. There is some question about why memory deteriorates, and there could be cohort effects [41], and visual perception and motor impairment could also play a role. However, one clear issue is that dementia incidence doubles with every 5 years of age after age 60, an exponential function [42], rising faster than mortality, a finding which persists after age 90 [43]. An exponential function is consistent with the Gompertz law of mortality [44, 45] and with an exponential increase in the rate of failure with age [46]. Thus, of specific interest is detecting and measuring the exponential increase of the rate of failure with age, which can be estimated by considering the trajectory of aging in the younger age-ranges through older ages. Though many of the older individuals had a total number of incorrect responses and recognition times that were in the ranges of young individuals, the data indicate a near linear but small increase of memory deterioration, both error rate and slowing of response time, with age between 21 and 75 years of age (when the exponential component is slight), transitioning to a progressively increasing rate of deterioration after age 75, consistent with the expected exponential factor which dominates the trajectory in this age range.

A central issue is to understand the factors which explain the observed deterioration of memory function with age. One major contributor is the progressive development of AD pathology with age [47]. However, there are many other health conditions which contribute to progressive failure of brain function with increasing age. While the data for more elderly subjects suggests that an exponential increase of memory failure is occurring, still, there are many very elderly individuals who are performing within the normal range for younger individuals. There is also known variability in the genetic factors associated with AD [48, 49], and likely numerous other complex factors leading to the deterioration of memory with age. Such factors allow for an increase of variability of performance with age and for some older individuals to have better performance.

### Screening for memory impairment, dementia, and AD

An important area is the use of memory testing to screen for clinically significant impairment of cognitive function, potentially indicative of dementia and AD [6, 31, 50–52]. The CCRT presented here can be done in a short amount of time, provides two important metrics of memory performance, incorrect percentage and recognition time, and has minimal cost. However, for clinical acceptance, there should be a “cost-worthiness” analysis [6, 50] for determining test benefit. Further, there is the important issue of selecting the optimal cut-point, balancing sensitivity and specificity [6, 53] for indicating the percent incorrect to be too high or the recognition time to be too slow, so optimally indicating the presence of cognitive impairment. The underlying problem is that dementia in general, and particularly AD, develop on a continuum over a long period of time, going from the broad normal range to obvious cognitive impairment. The measurement precision of the CCRT suggests that this test could serve as a highly useful screening test for cognitive impairment, dementia, and AD. Since the CCRT can be repeated frequently, this approach can potentially provide a baseline and could indicate clinically relevant change over time.

Related to screening, an important issue is how early before onset of dementia (or even subtle cognitive impairment) a clinically relevant deficit can be detected. Early studies modelling the time-course of cognitive change in AD suggested that the first cognitive impairment associated with the development of dementia occurred 4–6 years before the criteria for dementia are met [9, 33, 54–56]. More precise tests predict conversion to dementia after 10 years [34, 57]. Careful analysis of neurofibrillary changes in the brain at autopsy trace the onset of AD back to over 40 years before the onset of dementia [47, 58], and with the development of imaging of amyloid-β in the brain and cerebrospinal fluid measures of amyloid-β, there are AD-related changes measurable in living individuals at least 20 years before dementia, which are also highly related to APOE genotype [59]. It has yet to be determined which of these two substances might cause changes that can be detected earliest by advanced cognitive assessment. Within this progression, the data from this study suggest that this highly precise measure of EM function, could detect very early changes associated with AD potentially 10 years before the diagnosis of dementia, and possibly even much earlier, particularly with multiple testing repeated over an extended period. AD is a condition that develops across the full life-span of an individual, consistent with its predominant genetic basis [44, 48], and it is important to consider the long preclinical AD condition [60, 61] in trying to determine the earliest cognitive markers of early neurodegeneration [62].

### Screening for mild cognitive impairment (MCI)

As AD has been better understood, it has become clear that the attack of the AD pathological process in the brain specifically affects mechanisms of neuroplasticity [63–67], explaining the distribution of AD neuropathology [68] and the disruption of EM [1, 69]. The disruption of EM which precedes the dementia of AD has been given the clinical term “MCI due to AD”, similar to “prodromal AD”, with diagnostic criteria including a “concern regarding a change in cognition  ...   and impairment in one or more cognitive domains, with an impairment in EM most commonly associated with AD, with scores on cognitive tests 1–1.5 STDs below the mean for age ...  ” [70]. Several assessment approaches have been examined as screening tests for MCI; most focusing on memory testing [11, 71–74], as well as for making a diagnosis of MCI [7, 23, 75–77]. However, the MCI diagnosis is crude and not well implemented clinically. The specific statistical calculations of the standard deviations of the CCRT presented here could be of considerable practical utility in identifying individuals meeting this definition for MCI or defining which individuals have cognitive impairment consistent with early AD.

With progressively increasing attention to MCI, more precise assessment technologies are being developed for when an individual lies on the temporal trajectory from normal to dementia [55, 76, 78, 79], and such improved approaches are being suggested for measuring the progressive decline of cognitive function associated with early AD as well as assessing the benefits of intervention trials [13–15, 75, 80].

### Statistical issues in recognizing memory impairment

Since the critical issue in pre-clinical AD is the assessment of EM function, the CCRT presented here appears to be a potentially valuable choice for measuring EM, offering high-precision of two EM components, memory failure rate and memory access slowing, with clear age normative reference values. This test has such precise metrics with respect to age, that the 1 standard deviation metric for age 65 years—over 10% incorrect responses (5 errors) or slower than 1.1 s—could be used as a cut-point for supporting an MCI diagnosis. Since there is not a “speed-accuracy” trade-off for the population, but a small positive relationship between these metrics, either metric, error rate or recognition time, could be considered for screening purposes. Further, for the precise evaluation of change with age, the CCRT allows a determination of significant clinical variation to be made with respect either to a standard adult performance or a common age-related decline. While more data is needed to validate this CCRT for clinical purposes, in general, conditions such as AD develop very slowly over time in normal individuals, and a metric as clear as the CCRT can help to determine when a specific condition has developed. Further, following the performance of an individual over time may provide a more sensitive metric of developing impairment.

One STD below the average performance could be useful for estimating a problem in certain contexts, such as the existence of a concern for the individual’s cognitive function. However, for individuals who perform below 2 STD for age, a common level for developing a clinical concern on a laboratory test, these data suggest that the CCRT performance should be an alert for a clinical concern. Further study of the statistical cut-offs for this CCRT with respect to clinical problems is warranted.

### Issues for clinical use of online cognitive assessment

Several computerized assessment packages for cognition assessment have been developed, though most are based on translations of traditional neuropsychological tests to computer administration [8, 16–19, 80–82]. Computerized testing has been shown to be well tolerated by elderly individuals and is clinically useful [32], though impaired performance is accompanied by increased distress [83]. The present test appears to have been well tolerated by those who tried it, and many individuals took the test repeatedly, suggesting that this CCRT is engaging and not onerous as are many traditional memory tests.

While this study clearly shows the distribution of errors and response times in this sample of individuals, there is no absolute confidence in the information, even age and gender, about the subjects whose performances were analyzed in this report. With respect to clinical conditions, there is no direct knowledge of any clinical conditions of the subjects, cognitive, sensory, motor, metabolic, etc., which may have affected performance or performance distributions. Also, subjects may have participated in this study because of such concerns, which would skew the presented measurements. Further, none of the data reported here provide any validation of this CCRT for diagnosis of any clinical condition. Even the confidence in the calculated distributions cannot be assumed to be free of contamination by any factor which might be controlled in a face-to-face evaluation. Accordingly, more testing of this CCRT, optimally in a controlled environment with a defined population, is needed for validation. However, given the reasonable performance levels of over 97% of the individuals adhering to the performance requests of the test, online testing of memory functioning is a promising approach for the future.

### Conclusions

This report addresses the major need for the development of a short, efficient test for assessment of EM [84]. A particular application, which was the original intent for the development of this test, was detecting the early cognitive changes associated with AD and using of changes in these measures to track the early AD course more precisely, for clinical monitoring and therapeutic investigation. This CCRT, testing the exact aspect of EM most related to neuroplasticity and AD, seems to be the reasonable approach for assessing the cognitive impairment that precedes the dementia associated with AD and accompanies the first phases of this dementia. Moreover, EM impairment is impaired in numerous conditions in addition to dementia, such as traumatic brain injury, hypoxic or hypoglycemic brain damage, stroke, various vitamin and hormone deficiencies, and effects of toxic substances, particularly alcohol and marijuana, but also the effects of anesthesia and cancer chemotherapy. This short, precise test could be of great utility for assessment in all such conditions. Further, this CCRT, using a large set of complex pictures, can be repeated frequently, with multiple forms, even several times per day, without subject frustration, to assess status and change over time and increase the confidence in the performance measurements. This study clearly demonstrated a feasible approach to quantifying memory function, specifically EM, which could have wide-spread applicability. Further, the CCRT described here, has the potential for this test to be used repeatedly in individuals to assess EM change over time, either progression or even improvement.
